# Endogenous epitope tagging of a JAK/STAT ligand Unpaired1 in *Drosophila*

**DOI:** 10.17912/micropub.biology.000387

**Published:** 2021-08-31

**Authors:** Masahiko Takemura, Yi-Si Lu, Eriko Nakato, Hiroshi Nakato

**Affiliations:** 1 Department of Genetics, Cell Biology, and Development, University of Minnesota, Minneapolis USA

## Abstract

Unpaired1 (Upd1) is a ligand of the Janus kinase/signal transducer and activator of transcription (JAK/STAT) signaling pathway in *Drosophila*. In this study, using the CRISPR/Cas9 technique, we generate a transgenic fly strain in which a hemagglutinin (HA) epitope tag sequence is inserted into the endogenous locus of the *upd1* gene. Anti-HA antibody staining confirms that the distribution of the epitope-tagged Upd1::HA in various tissues is consistent with *upd1* expression patterns revealed by previous studies. This transgenic fly strain will be useful in studying the expression, localization, and association partners of Upd1, and thus will contribute to understanding how activation of the JAK/STAT pathway is regulated.

**Figure 1. Epitope tagging of endogenous Upd1 using CRISPR/Cas9-mediated homologous recombination:**
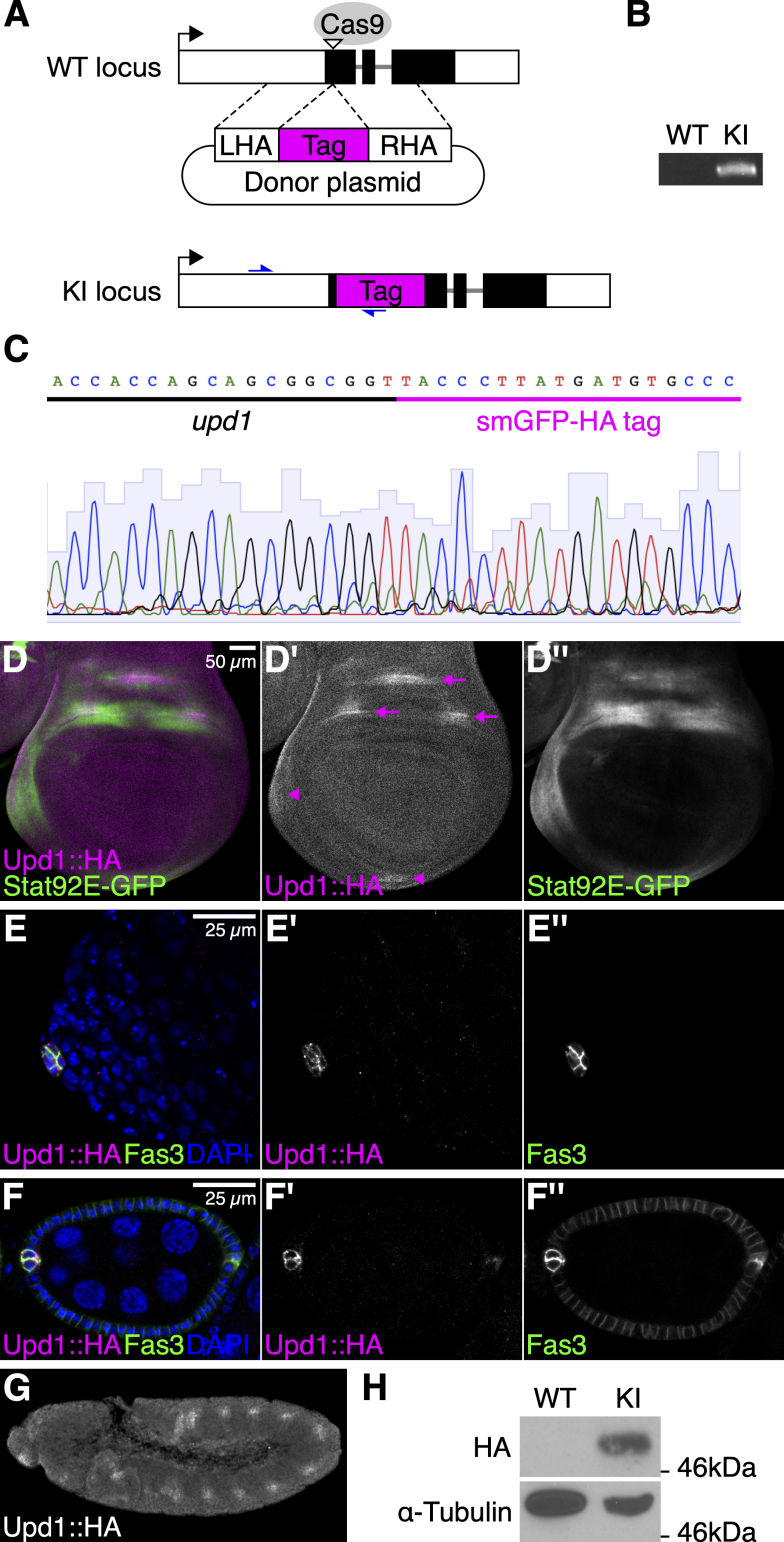
(A) Schematic illustration of the knock-in strategy of the non-fluorescent smGFP-HA sequence into the endogenous *upd1* locus using the CRISPR/Cas9 technique. The black boxes indicate the coding sequence of *upd1*. The open triangle shows the Cas9 target site. The magenta box indicates the smGFP-HA tag. LHA, left homology arm; RHA, right homology arm. (B) PCR analysis using genomic DNA isolated from wild-type (WT) and *upd^KI.HA^* (KI) flies. A DNA fragment was amplified using primers (indicated by blue arrows in A) in *upd^KI.HA^*, whereas the band is absent in WT. (C) Sanger sequencing of the PCR product from *upd^KI.HA^* genomic DNA for confirming precise tag integration. (D–G) The expression of HA-tagged Upd1 (Upd1::HA) detected by an anti-HA antibody in the third-instar larval wing disc (D–D”), the adult testis apex (E–E”), the stage 6 egg chamber of the adult ovary (F–F”), and the stage 11-12 embryo (G). (D–D”) In the wing disc, the Upd1::HA expression (magenta) is observed within the presumptive dorsal wing hinge (arrows) and the body wall (arrowheads). The Stat92E-GFP expression (green) is detected in surrounding cells. (E–E”) In the testis, the expression of Upd1::HA (magenta) is observed specifically in the hub cells, which are marked by Fas3 (green). (F–F”) In the ovary, the expression of Upd1::HA (magenta) is observed in two pairs of polar cells, which are marked by Fas3 (green). Nuclei are shown in blue (E and F). (G) In the embryo, Upd::HA is detected in the tracheal pits. (H) Western blots of WT and *upd^KI.HA ^*embryonic extracts probed with anti-HA and anti-α-Tubulin antibodies.

## Description

The Janus kinase/signal transducer and activator of transcription (JAK/STAT) signaling pathway is involved in various biological processes, including cell proliferation, differentiation, and apoptosis (Morris *et al.* 2018). The core components of the JAK/STAT pathway are evolutionarily conserved between humans and flies (Arbouzova and Zeidler 2006). While more than 50 cytokines activate the JAK/STAT pathway in humans, there are only three JAK/STAT ligands in *Drosophila* (Morris *et al.* 2018). Unpaired 1 (Upd1), one of the three ligands of the *Drosophila* JAK/STAT pathway, is involved in many developmental processes, including growth and patterning of the wing disc (Ayala-Camargo *et al.* 2013; Recasens-Alvarez *et al.* 2017), stem cell maintenance in the testis (Cuevas and Matunis 2011), and specification of follicular epithelial cell fates in the ovary (McGregor *et al.* 2002).

Since Upd1 interacts with the extracellular matrix (Harrison *et al.* 1998; Hayashi *et al.* 2012), it is essential to understand its distribution and association partners at a protein level using antibodies. Polyclonal antibodies against Upd1 have been generated previously (Harrison *et al.* 1998; Zhang *et al.* 2013; Beshel *et al.* 2017). However, since polyclonal antibodies are typically purified from the serum of the immunized animal, their supply is limited and exhaustible.

Epitope tagging of endogenous protein is an alternative effective means for studying the expression, localization, and interactive association of a protein under normal and pathological conditions. Once an epitope tag sequence is successfully inserted into an endogenous gene locus, the tagged protein can be readily detected or purified using a commercially-available well-characterized monoclonal antibody for the epitope. In this study, we generated a knock-in strain in which an epitope tag is inserted into the endogenous locus of *upd1* using CRISPR/Cas9-mediated homology-directed repair.

Upd1 has multiple potential furin cleavage sites (Arg133, Lys332, Arg402, and Arg405), which are predicted by the ProP v.1.0 server with relatively higher scores (≥0.45; Duckert *et al.* 2004). We inserted the non-fluorescent GFP-like protein containing 10 copies of influenza hemagglutinin (HA) epitope tag (smGFP-HA; Viswanathan *et al.* 2015) after Gly35 of Upd1, downstream of the N-terminal signal sequence (Fig. 1A). Flies with tag integration were screened by PCR using a tag-specific and genomic primer pair (Fig. 1B). Sanger sequencing was performed for validating precise integration (Fig. 1C). Here, we refer to this transgenic strain as *upd1^KI.HA^* and its protein product as Upd1::HA. The *upd1^KI.HA^* flies are viable and fertile.

We examined the expression patterns of the HA-tagged Upd1 from the *upd1^KI.HA^* flies in the wing disc, ovary, testis, and embryo using an anti-HA antibody. In the wing disc, we found that Upd1::HA was expressed within three regions of the presumptive dorsal wing hinge as well as in the anterior and ventral body wall (Fig. 1D). This observation is consistent with a previously-reported result determined by *in situ* hybridization (Johnstone *et al.* 2013). We also confirmed that using a Stat92E-GFP reporter (Bach *et al.* 2007), the JAK/STAT pathway was activated in surrounding regions of the Upd1::HA-expressing cells (Fig. 1D). Similarly, the expression of Upd1::HA was detected in a group of quiescent somatic niche cells (called hub cells) in the adult testis (Fig. 1E) and two pairs of polar cells at each end of the egg chamber in the adult ovary (Fig. 1F), as previously reported (Silver and Montell 2001; Tulina and Matunis 2001; Beccari *et al.* 2002). In the stage 11-12 embryo, we confirmed that Upd1::HA is expressed in the tracheal pits, consistent with the previously-reported pattern of *upd1* mRNA (Fig. 1G; Harrison *et al.* 1998). Western blotting of embryonic extracts found that Upd1::HA was detected as a band at around 50kDa (Fig. 1H). This band size is consistent with the cleavage at Arg133, although biochemical analysis is required to determine the precise cleavage site. Taken together, these results demonstrate that the expression of Upd1 is faithfully detected using this *upd1^KI.HA^* fly strain. This strain will also be useful in studying the localization and association partners of Upd1, and thus will contribute to future research on the JAK/STAT pathway.

## Methods

***Drosophila* strain construction**

The *upd1^KI.HA^* fly strain was generated using CRISPR/Cas9-mediated gene editing as previously described (Gratz *et al.* 2014; Takemura *et al.* 2021). Briefly, to construct a single guide RNA (sgRNA) plasmid, 5′-CTTCGAACCGTTAGACCGCCGCTGC-3′ and 5′-AAACGCAGCGGCGGTCTAACGGTTC-3′ were annealed and ligated in the *Bbs*I-digested pU6-BbsI-chiRNA plasmid (a gift from Melissa Harrison, Kate O’Connor-Giles, and Jill Wildonger; Addgene #45946). We constructed a donor plasmid containing 757-bp and 975-bp arms homologous to sequences in the *upd1* gene, flanking smGFP-HA and a Gly-Gly-Ser linker. A mixture of 50 ng/µl of the sgRNA plasmind and 250 ng/µl of the donor plasmid was injected into the *vasa-Cas9* embryos by BestGene Inc. The *upd1^KI.HA^* allele was screened by PCR and verified by Sanger sequencing.

**Immunohistochemistry**

Dissection and antibody staining were performed as previously described (Takada *et al.* 2015; Takemura *et al.* 2020). The following primary antibodies were used: rabbit monoclonal anti-HA C29F4 (1:1000, Cell Signaling Technology, #3724) and mouse monoclonal anti-Fas3 7G10 (1:50, Developmental Studies Hybridoma Bank [DSHB], deposited by Corey Goodman). Alexa548- and Alexa633-conjugated secondary antibodies (Thermo Fisher Scientific) were used at a dilution of 1:500. Nuclei were stained with 1 µg/ml DAPI (Thermo Fisher Scientific, #62248). Images were acquired on an LSM710 confocal microscope (Carl Zeiss).

**Western blotting**

Embryonic protein extraction and western blotting were performed as previously described (Kleinschmit *et al.* 2013; Takada *et al.* 2015). The following antibodies were used: rabbit monoclonal anti-HA C29F4 (1:1000, Cell Signaling Technology, #3724), mouse monoclonal anti-α-Tubulin DM1A (1:1000, Sigma-Aldrich), anti-rabbit HRP-linked IgG (1:10000, Cell Signaling Technology) and anti-mouse HRP-linked IgG (1:10000, Cell Signaling Technology).

## Reagents

The following fly stocks were used in this study:

**Table:** 

**Strain**	**Genotype**	**Available from**
*vasa-Cas9*	y[1] M{GFP[E.3xP3]=vas-Cas9.RFP-}ZH-2A w[1118]	Bloomington Drosophila Stock Center (BDSC) #55821
*Stat92E-GFP*	w[1118]; P{w[+mC]=10XStat92E-GFP}1	BDSC #26197
*upd1^KI.HA^*	upd1[KI; smGFP-HA]	This study
